# Supplementary material for: Multiphase superconductivity in PdBi2
Source: Nat Commun. 2025 Jan 2;16:291. doi: 10.1038/s41467-024-54867-x (PMC11696495; doi:10.1038/s41467-024-54867-x)
Supplement: Supplementary file 1 — Supplementary Information [file 41467_2024_54867_MOESM1_ESM.pdf]

SUPPLEMENTARY INFORMATION

Multiphase superconductivity in $PdBi_2$

Lewis Powell et al.

1. Supplementary Figures

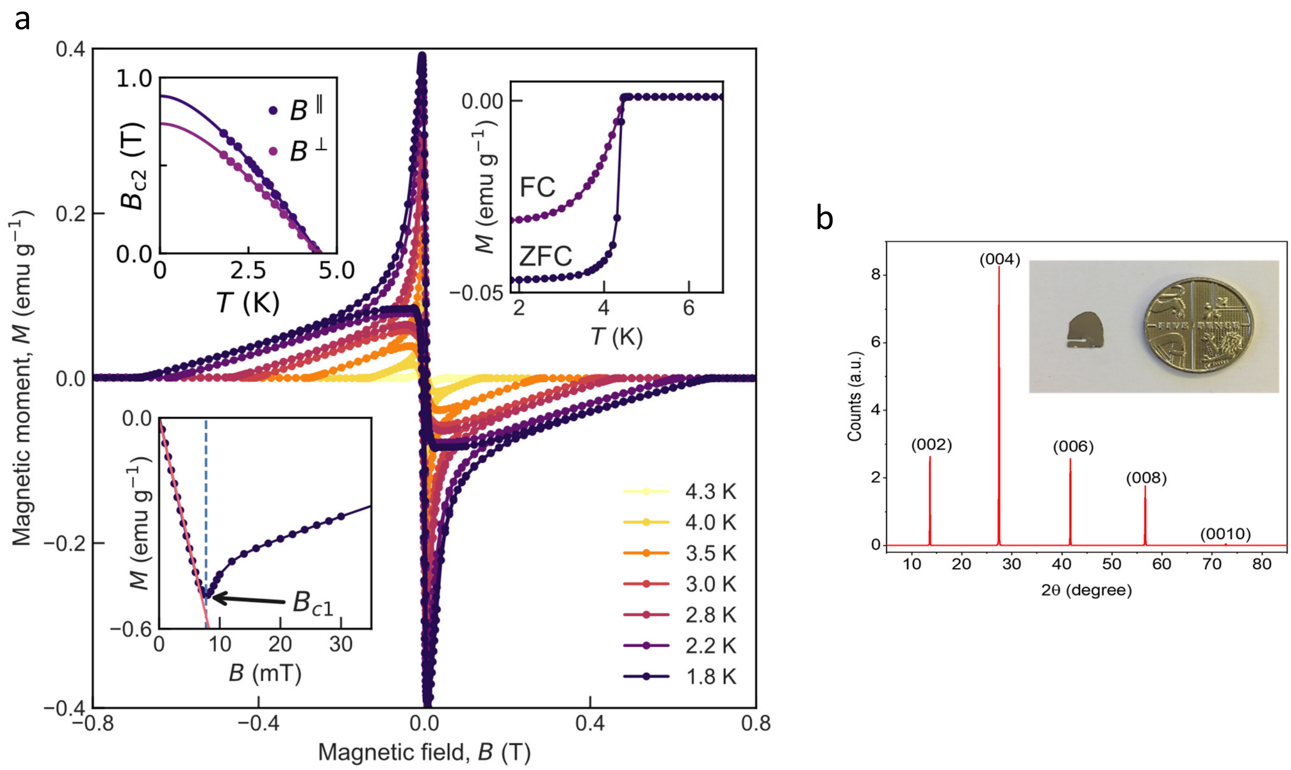

Supplementary Figure 1 | Characterization of bulk β-$PdBi_2$ crystals. Typical magnetization curves for bulk samples at different temperatures. Shown are data for a ~100μm thick crystal. **a**, *Main panel:* Magnetisation vs applied magnetic field at several temperatures (see legend). *Lower left inset:* Zoom of the magnetisation curve at 1.8 K corresponding to vortex penetration at the lower critical field B_{c1}. *Upper left inset*: Temperature dependence of the upper critical field for in-plane and out-of-plane orientations of the applied B, see legend. Solid lines are fits to WHH theory. *Top right inset*: Superconducting transition as seen in dc magnetization of a ~100 μm thick crystal; $T_c = 4.5$ K. Shown are field-cooling (FC) and zero-field cooling (ZFC) magnetization curves. **b,** X-ray diffraction pattern of our single crystals showing sharp (00l) peaks (FWHM 0.03°), accurately matching the β-phase of $PdBi_2$. The inset shows a typical flat section of the as-grown crystal used to exfoliate crystals for device fabrication**.**

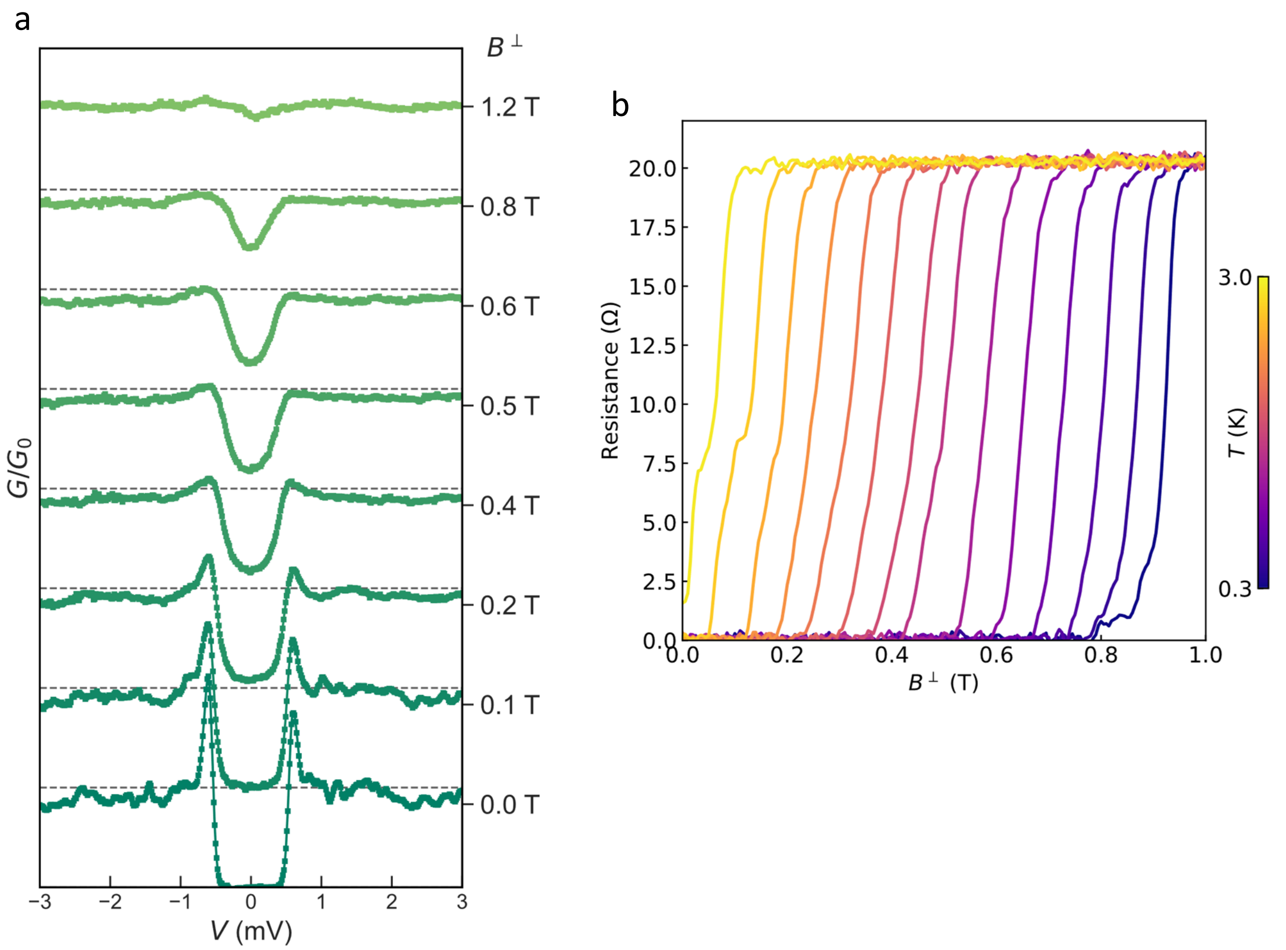

Supplementary Figure 2 | Evolution of the tunnelling spectra and device resistance in out-of-plane magnetic field. a, Tunnelling spectra measured at T = 0.3K as a function of perpendicular field, $B^{\perp}$, see labels for field values. Data for device C. The spectra cannot be translated directly into the superconducting DoS due to an (unknown) contribution from the normal cores of vortices that penetrate the junction in this field range. **b**, Resistance R as a function of the out-of-plane field $B^{\perp}$, measured at different temperatures. Data for device A.

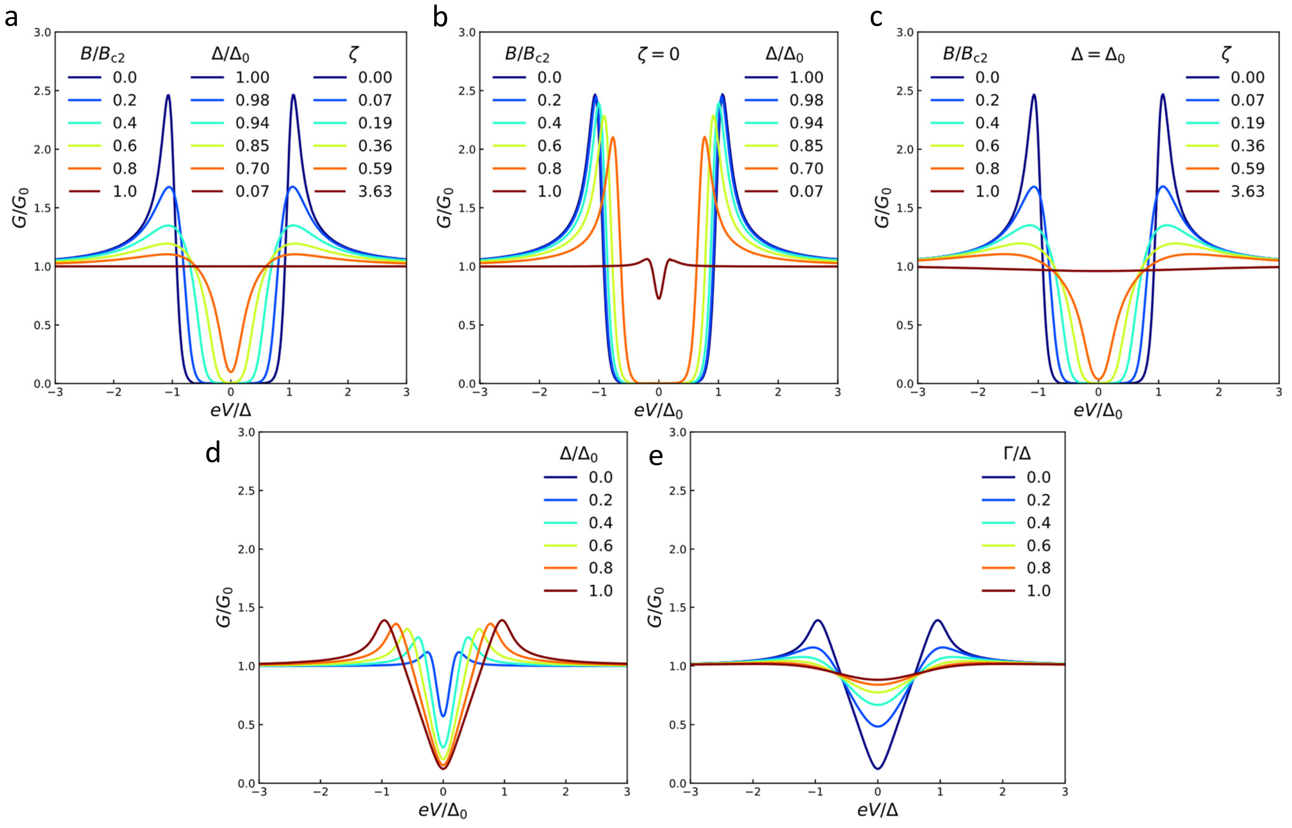

Supplementary Figure 3 | Modelling of the tunnelling spectra expected for *s*-wave and nodal *p*-wave superconductors in magnetic field. a, Evolution of the conductance spectra for an s-wave superconductor as a function of two parameters, order parameter Δ and pair-breaking strength ζ. The spectra are calculated using eqs. (9)-(12) in Methods (Maki theory). Legends show ζ and Δ computed for selected values of B/B_{c2} using eqs. (11),(12). The spectra demonstrate qualitative features of the effect of magnetic field as described in the main text: zero-bias conductance (ZBC) remains zero and the spectra are fully gapped up to $B{\sim}0.7B_{\mathrm{c2}}$; quasiparticle peaks are almost fully suppressed at $B > 0.5B_{\mathrm{c2}}$. **b,** Same as (a) but only the order parameter Δ is allowed to vary, while $\zeta = 0$ for all B (see legends). This demonstrates that decreasing Δ changes the scale but not the shape of the spectra, except very close to the transition to the normal state, where thermal broadening becomes important. The relative insensitivity of the spectral shape to increasing B in this case follows from the Ginzburg-Landau dependence $\Delta(B){\sim}\sqrt{(1 - B/B_{\mathrm{c2}})^2}$, such that Δ is only suppressed close to B_{c2}. **c,** Same as (a) but with the order parameter fixed at $\Delta = \Delta_0$. Comparing (a) and (c) shows that the evolution of the spectral shape is mainly driven by the pair-breaking strength ζ: it reduces the gap and strongly suppresses the quasiparticle peaks, while ZBC remains zero for all but the largest values of ζ>1. **d-e,** Calculated evolution of the conductance spectra for a p-wave superconductor characterised by an order parameter with line nodes, eq. (3) in the main text. Panel (d) shows the effect of reducing Δ while the broadening parameter Γ is fixed at $\Gamma = 0$. Decreasing Δ changes the slope of the linear DOS inside the gap but has a weak effect on ZBC. Panel (e) shows the effect of an increasing Γ (equivalent to pair-breaking strength in the Maki model). Increasing Γ has a strong effect on the ZBC. In all panels the temperature is $T = 0.1\Delta/1.76k_{\mathrm{B}} = 0.1T_{\mathrm{c}}$, corresponding to experiment ($T = 0.3$K, $T_{\mathrm{c}} \approx 3$ K).

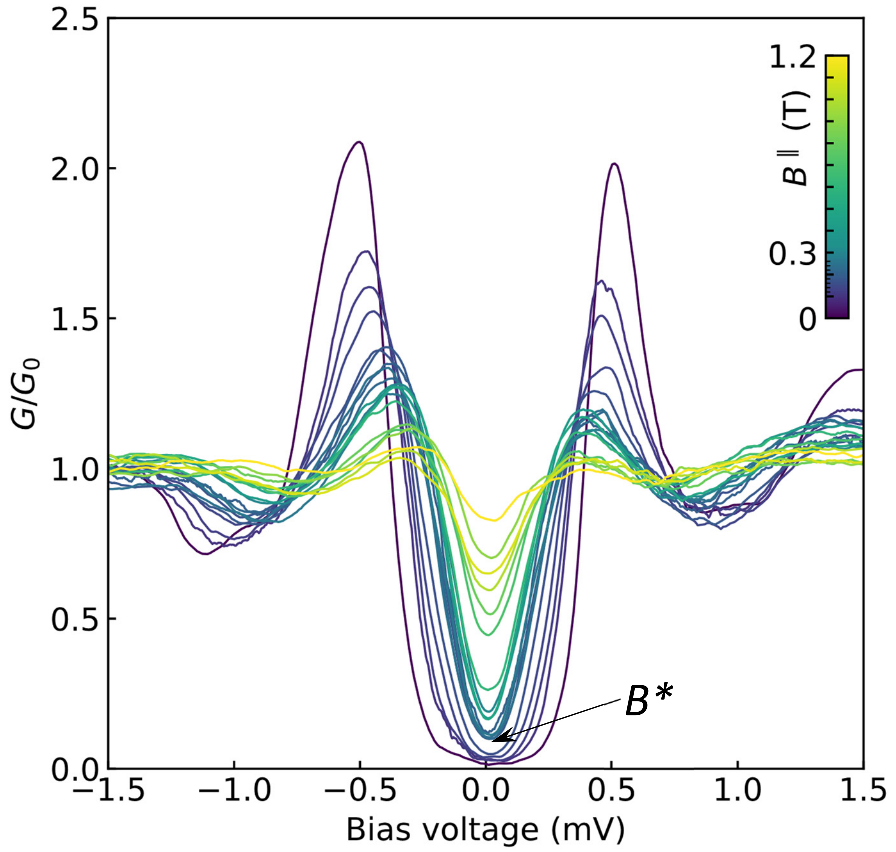

Supplementary Figure 4 | Evolution of the tunnelling spectra for in-plane magnetic field. Measured spectra are shown without a shift, to emphasise the change in spectral shape at B^*. Data for device B; $T = 0.3$K.

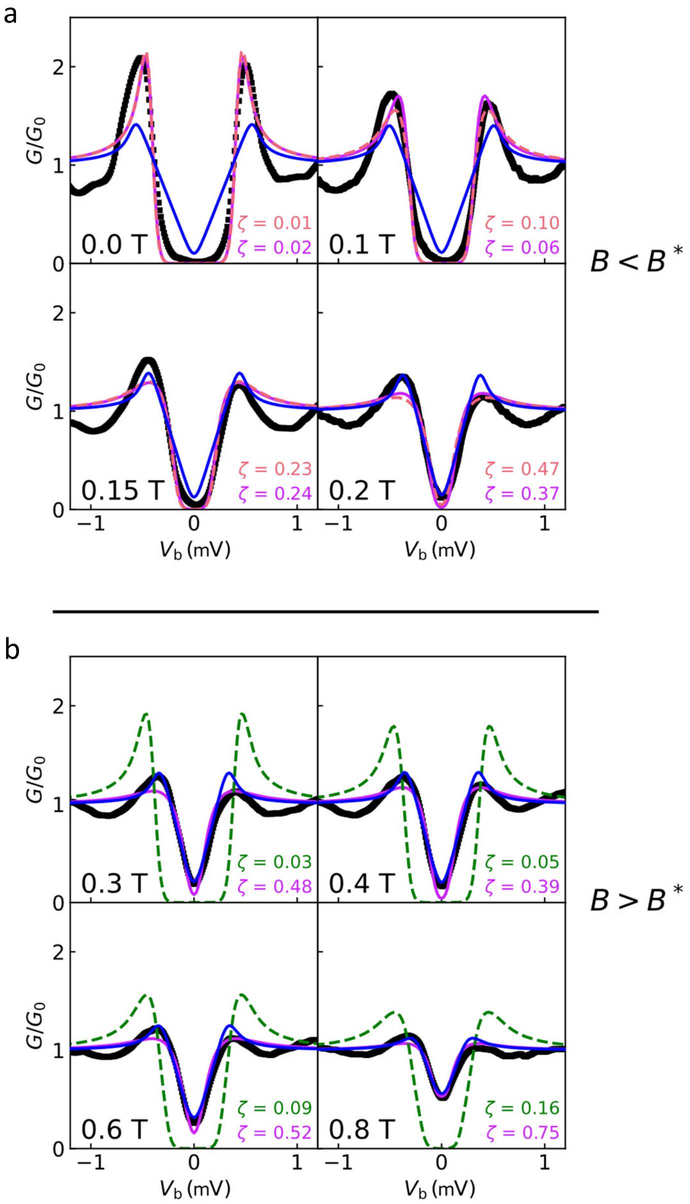

Supplementary Figure 5 | Analysis of the tunnelling spectra under in-plane magnetic field and comparison with theoretical models for *s*-wave and *p*-wave superconductivity. a, Representative tunnelling spectra for in-plane $B^{\parallel}$ below the transition field B^*, and comparison to the two theoretical models. Black symbols are data, magenta solid lines are best fits to the Maki theory with Δ and the pair-breaking ζ used as fitting parameters, and red dashed lines are spectra *calculated* from the Maki theory using $\Delta_0 = 0.44$ meV (as obtained from fitting at $B = 0$) and $B_c^{s-\mathrm{wave}} = 0.25$T extrapolated from $\Delta(B^{||})$ below B^* in Fig. 3a in the main text. Color-coded legends show corresponding values of the pair-breaking strength (calculated and obtained from the fit, respectively). Fitted and calculated spectra are practically indistinguishable and both accurately describe an *s*-wave superconductor (compare with panel (a) in Supplementary Fig. 3). In contrast, fits to a nodal gap (blue solid lines) are poor, except very close to B^*. **b,** Same for the spectra measured at $B^{\parallel} > B^*$. Green dashed lines show spectra that would be expected from the Maki theory for a 'global' upper critical field B_{c2}=1.6 T (where $PdBi_2$ transitions to the normal state). Solid blue lines are best fits to the nodal model. For completeness, we also attempted fitting with the Maki model by allowing the pair-breaking strength to take on arbitrarily large values. These fits are shown by solid magenta lines and the corresponding values of the pair-breaking strength are shown by color-coded legends. It is clear that the spectra for all $B > B^*$ are best described by the nodal DoS.

Superficially, the Maki model also describes the spectra reasonably well but requires pair-breaking strength well beyond the values predicted by this theory – the latter are shown in the legends in green. We therefore conclude that the Maki model is not valid in this field range, while the assumption of the p-wave order parameter with line nodes provides an accurate fit to the experimental data.

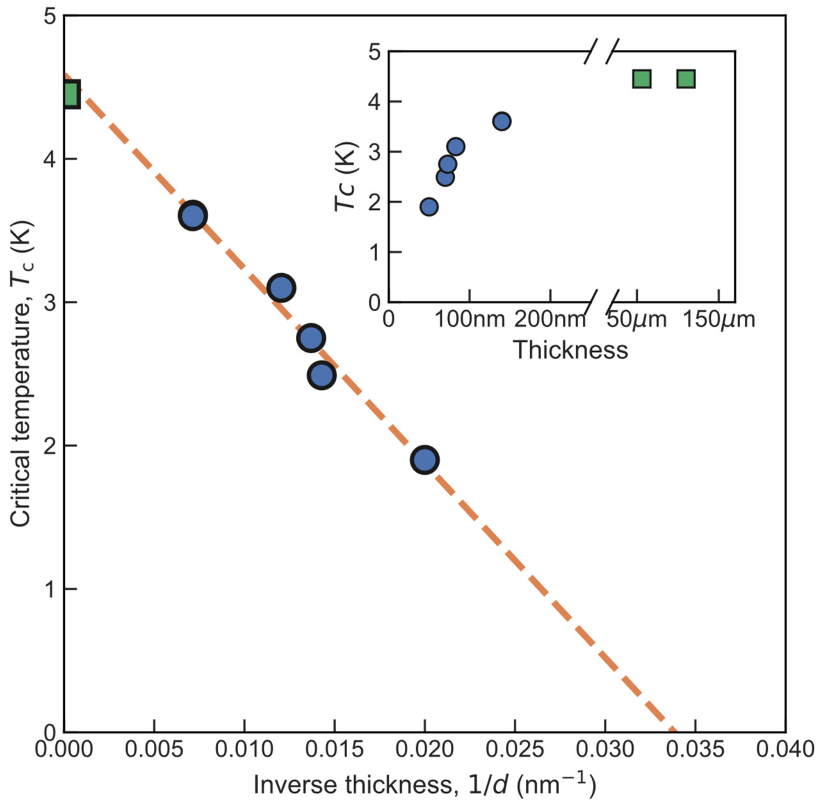

Supplementary Figure 6 | Dependence of the transition temperature T_c on the thickness of $PdBi_2$ crystals. *Main panel:* Critical temperatures for different samples follow a $1/d$ dependence, where d is the crystal thickness. Blue circles are data obtained from $R(T)$ measurements on different tunnelling devices; green squares are data obtained from magnetisation measurements on bulk crystals (d >10µm). Inset shows the same data on linear scale.

2. Supplementary Notes

2.1. Density of states of a nodal superconductor

The low-energy excitation spectrum of a superconductor can be calculated from the Nambu-Gorkov Green's functions. In the absence of disorder and magnetic field the diagonal and off-diagonal parts are[10]

$$G(\boldsymbol{k}, i\epsilon_n) = \frac{i\epsilon_n + \xi_{\boldsymbol{k}}}{(i\epsilon_n)^2 - \xi_{\boldsymbol{k}}^2 - |\Delta_{\boldsymbol{k}}|^2}, \tag{S1}$$

$$F(\boldsymbol{k}, i\epsilon_n) = \frac{\Delta_{\boldsymbol{k}}^\dagger}{(i\epsilon_n)^2 - \xi_{\boldsymbol{k}}^2 - |\Delta_{\boldsymbol{k}}|^2}. \tag{S2}$$

Here, $\xi_{\boldsymbol{k}} = \frac{k^2}{2m}$ is the band energy and $\epsilon_n = \frac{(2n+1)\hbar\pi}{k_B T}$ the fermionic Matsubara frequency. For a p-wave superconductor with an order parameter $\Delta_{\hat{\boldsymbol{k}}} = \Delta\cos(\theta_{\boldsymbol{k}})$ the normalized DoS is given by

$$\begin{aligned} \frac{N_S(E,\Delta)}{N_0} &= -\frac{1}{N_0}\mathrm{Im}\int_0^\infty \frac{dkk^2}{2\pi}\int\frac{d\Omega_{\boldsymbol{k}}}{4\pi} G(\boldsymbol{k}, i\epsilon_n)\bigg|_{i\epsilon_n\to E+i\eta} \\ &= -\mathrm{Im}\int\frac{d\Omega_{\boldsymbol{k}}}{4\pi}\int_{-\infty}^{\infty} d\xi\, \frac{i\epsilon_n+\xi_{\boldsymbol{k}}}{(i\epsilon_n)^2-\xi_{\boldsymbol{k}}^2-|\Delta_{\boldsymbol{k}}|^2}\bigg|_{i\epsilon_n\to E+i\eta} \\ &= \mathrm{Re}\int\frac{d\Omega_{\mathbf{k}}}{4\pi}\frac{E}{\sqrt{E^2-\Delta^2\cos^2(\theta)}} \\ &= \mathrm{Re}\left[\frac{E}{\Delta}\arcsin\left(\frac{\Delta}{E}\right)\right], \end{aligned} \tag{S3}$$

where $\Omega_{\boldsymbol{k}}$ is the solid angle spanned by the 3D unit vector $\hat{\boldsymbol{k}}$ and N_0 is the normal-state DoS. This expression is linear for $E < \Delta$ and gives rise to sharp but finite peaks at $E = \Delta$. The pair-breaking effect of the magnetic field and/or disorder can be included by replacing $E \to E + i\Gamma$ (see 'Fitting tunnelling data' in Methods) which yields

$$\frac{N_S(E,\Gamma,\Delta)}{N_0} = \mathrm{Re}\left[\frac{E+i\Gamma}{\Delta}\arcsin\left(\frac{\Delta}{E+i\Gamma}\right)\right]. \tag{S4}$$

2.2. Tight binding model

As shown in Supplementary Fig. 7, the crystal lattice of β-$PdBi_2$ is composed of covalently bonded trilayers held together by van der Waals forces. Each trilayer consists of an inner Pd square monolayer (grey atoms in Supplementary Fig. 7a) enclosed by two AA-stacked (atom-above-atom) square Bi monolayers (purple atoms in Supplementary Fig. 7a). We consider the two Bi monolayers within a trilayer as "sublayers" and label them with an index σ. Neighbouring trilayers are shifted such that the Bi atoms in one layer are lined up with the Pd sites in the other. Thus, the tetragonal crystal unit cell (black solid lines in Supplementary Fig. 7a) consists of six atoms, two Pd and four Bi. We now build three model Hamiltonians of different complexities:

- **I.** A 2D tight-binding model for a single Bi bilayer.
- **II.** A 3D tight-binding model built by stacking bilayers of model **I**, which allows comparing the results with literature.
- **III.** A 2D continuum model simplified from **I**, from which the superconducting gap equations used in this work are derived.

The 2D tight-binding model contains all symmetry-permitted hopping amplitudes between Bi sites up to next-nearest neighbours. The states that contribute to the Fermi surface are predominantly Bi p orbitals where hopping occurs between nearest- and next-nearest-neighbour atoms of each Bi bilayer (orange and green arrows in Supplementary Fig. 7a, respectively), as well as between Bi atoms of different bilayers (blue arrows in Supplementary Fig. 7a). These p-orbitals form intra-sublayer 'sigma' bonds with strength w and 'pi' bonds with strength δ. The inter-sublayer sigma and pi bonds have strength w' and δ' respectively. The effect of Pd d orbitals is captured by renormalising the Bi hopping parameters. The crystal field, $\pm\gamma$, couples p_z orbitals to the radial component, p_r, of neighbouring $p_{x,y}$ orbitals in the same sublayer. On the other hand, crystal-field-induced coupling between the two sublayers is forbidden, as Bi atoms from the sublayers are related by inversion symmetry.

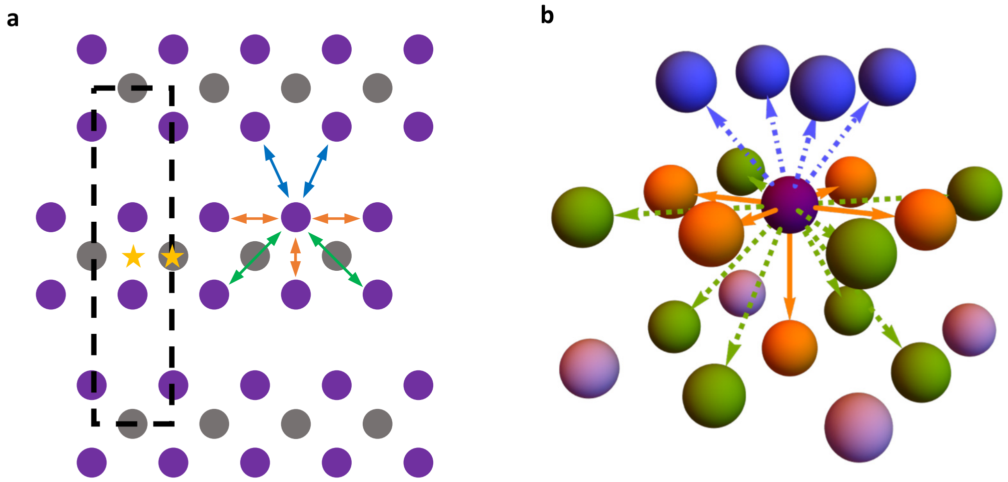

Supplementary Figure 7 | Crystal structure and tight-binding description of β-$PdBi_2$. a, Side view of the atomic arrangement. Two Bi and one Pd monolayers forming each trilayer are covalently bonded, while neighbouring trilayers are held together by weaker van der Waals forces. *Dashed rectangle*: the unit cell. *Yellow stars*: inversion centers. Arrows indicate hopping amplitudes used in the tight-binding calculations. **b,** 3D view of the crystal composed of Bi trilayers with atoms color-coded according to the hopping amplitudes (same color coding as in (a)).

We work in a 12-dimensional basis, $|p_i, \sigma, s\rangle$ where $p_i \in \{p_x, p_y, p_z\}$ is the orbital basis, $\sigma \in \{A, B\}$ is the sublayer index and $s \in \{\uparrow, \downarrow\}$ is the spin index. The Hamiltonian of the system contains nearest-neighbour (NN), next-nearest neighbour (NNN) and spin-orbit coupling (SOC) terms and reads

$$H = h^{\mathrm{NN}} + h^{\mathrm{NNN}} + h^{\mathrm{SOC}}. \qquad \text{(S5)}$$

Explicitly, the wavevector ($\boldsymbol{k}$)-dependent NN couplings are given by

$$\begin{aligned} h_{xx}^{NN} &= 2w\cos k_x - 2\delta\cos k_y - \delta', \\ h_{yy}^{NN} &= 2w\cos k_y - 2\delta\cos k_x - \delta'\sigma_x, \\ h_{zz}^{NN} &= -2\delta\left(\cos k_x + \cos k_y\right) + w'\sigma_x, \\ h_{xz}^{NN} &= (h_{zx}^{NN})^* = 2i\gamma\sin k_x\,\sigma_z, \\ h_{yz}^{NN} &= \left(h_{zy}^{NN}\right)^* = 2i\gamma\sin k_y\,\sigma_z, \end{aligned} \qquad \text{(S6)}$$

where the indexes $\alpha, \beta = x, y, z$ in $h_{\alpha\beta}^{NN}$ refer to the three p-orbitals and the Pauli matrices σ_x, σ_y and σ_z operate on the sublayer degree of freedom (terms that are not multiplied by a Pauli matrix are assumed to be proportional to the identity matrix). With a suitable choice of parameters w, w', δ, δ' and γ, the

bands $E(k_x, k_y)$ obtained from h^{NN} within this minimal model resemble the known band structure of β-$PdBi_2$ along Γ – X, but fails to reproduce the bands along Γ – M and M – X [1-4]. Additionally, this minimal model exhibits a symmetry between the energy spectra at Γ and M points, $E(0,0) = -E(\pi, \pi)$, which is not present in the DFT and ARPES results for β-$PdBi_2$ [1,2], implying that $h^{\rm NN}$ is missing essential terms.

The fact that the discrepancy lies in the direction of M = (π, π) indicates that the missing terms describe hopping with a diagonal component, most naturally arising from hopping to intralayer NNN sites. Since these have a comparable distance to next-nearest inter-sublayer neighbours, we include both hoppings in $h^{\rm NNN}$. The components of $h^{\rm NNN}$ are then given by

$$\begin{aligned} h_{xx}^{\rm NNN} &= 2(w'' - \delta'')\cos k_x \cos k_y + \big(a\cos k_x + b\cos k_y\big)\sigma_x, \\ h_{yy}^{\rm NNN} &= 2(w'' - \delta'')\cos k_x \cos k_y + \big(a\cos k_y + b\cos k_x\big)\sigma_x \\ h_{zz}^{\rm NNN} &= -4\delta''\cos k_x \cos k_y + c\big(\cos k_x + \cos k_y\big)\sigma_x, \\ h_{xy}^{\rm NNN} &= h_{yx}^{\rm NNN} = -2(w'' + \delta'')\sin k_x \sin k_y, \\ h_{xz}^{\rm NNN} &= (h_{zx}^{\rm NNN})^* = 2\sqrt{2}i\gamma''\sin k_x \cos k_y\,\sigma_z, \\ h_{yz}^{\rm NNN} &= (h_{zy}^{\rm NNN})^* = 2\sqrt{2}i\gamma''\sin k_y \cos k_x\,\sigma_z. \end{aligned} \tag{S7}$$

Here w'' and δ'' describe in-plane next-nearest neighbour hopping along diagonal intralayer π and σ bonds respectively. The parameters a, b and c denote hopping along some mixture of π and σ bonds between orbitals and their next-nearest inter-sublayer neighbours. Finally, γ'' denotes in-plane next-nearest neighbour diagonal hopping between $p_{x,y}$ and p_z atoms induced by the crystal field.

Both Hamiltonians $h^{\rm NN}$ and $h^{\rm NNN}$ are diagonal in spin. SOC can be included directly as $h^{\rm SOC} = \lambda \boldsymbol{L}\cdot\boldsymbol{S}$, where L_i are the usual orbital angular momentum operators acting on the orbital basis and $S_i = s_i/2$ are the spin operators. The high atomic number of Bi implies that the spin-orbit coupling λ is large in this system due to relativistic effects. Indeed, the atomic SOC of Bi is estimated to be ~0.5 eV [4].

2.3. 3D model

To compare our model with ARPES experiments and DFT calculations in the literature, we model a semi-infinite slab of β-$PdBi_2$ as a stack of square Bi bilayers with nearest-neighbour interactions by recursively coupling single-bilayer Green's functions and renormalising them into an effective surface slab. To this end we follow the efficient method of López Sancho et al[5] that describes the surface of a 2^N-layer stack at step N. We write the semi-infinite Hamiltonian as the tridiagonal matrix,

$$\mathcal{H} = \begin{pmatrix} H & V & & \\ V^\dagger & H & V & \\ & V^\dagger & \dots & \dots \\ & & \dots & \dots \end{pmatrix}, \tag{S8}$$

where we neglect momentarily the NNN part of the Hamiltonian; thus $H = h^{\rm NN} + h^{\rm SOC}$. Here, V is the interlayer hopping which takes the form

$$V = v e^{-\frac{i(k_x + k_y)}{2}}\sigma_-, \tag{S9}$$

where $\sigma_\pm = (\sigma_x \pm i\sigma_y)/2$ and v is a matrix in the orbital basis with the following elements:

$$\begin{aligned} v_{xx} = v_{yy} &= (2\,\sin^2\phi\,(w_v - \delta_v) - 4\cos^2\phi\,\delta_v)\cos\frac{k_x}{2}\cos\frac{k_y}{2}, \\ v_{zz} &= 4(\cos^2\phi\,w_v - \sin^2\phi\,\delta_v)\cos\frac{k_x}{2}\cos\frac{k_y}{2}, \end{aligned}$$

$$v_{xy} = v_{yx} = -2\sin^2\phi\,(w_v + \delta_v)\sin\frac{k_x}{2}\sin\frac{k_y}{2}, \quad \text{(S10)}$$
$$v_{xz} = v_{zx}^* = 2\sqrt{2}i\cos\phi\sin\phi\,(w_v + \delta_v)\sin\frac{k_x}{2}\cos\frac{k_y}{2},$$
$$v_{yz} = v_{zy}^* = 2\sqrt{2}i\cos\phi\sin\phi\,(w_v + \delta_v)\cos\frac{k_x}{2}\sin\frac{k_y}{2}.$$

Here ϕ is the interlayer stacking zenith angle and w_v and δ_v are new parameters for interlayer hopping. The corresponding semi-infinite Green's function $\mathcal{G}(E,\boldsymbol{k})$ is defined by

$$\big(E - \mathcal{H}(\boldsymbol{k})\big)\mathcal{G}(E,\boldsymbol{k}) = \mathcal{I}, \quad \text{(S11)}$$

where $\mathcal{I}$ is the identity operator. The surface spectral function takes the usual form

$$\mathcal{A}_S(E) = \mathrm{Tr}\,\mathrm{Im}\,G_S(\boldsymbol{k}, E - i\epsilon). \quad \text{(S12)}$$

The retarded surface Green's function, $G_S(\boldsymbol{k}, E - i\epsilon)$, can be obtained from the single-layer matrices H and V by iterating the decimation procedure described in ref. [5] until G_S converges to arbitrary precision. The calculated spectral function is shown in Fig. S3. The validity of our approximation is confirmed not just by the agreement of the bulk bands with the literature[1,6], but also by the presence of helical topological surface states[1,6].

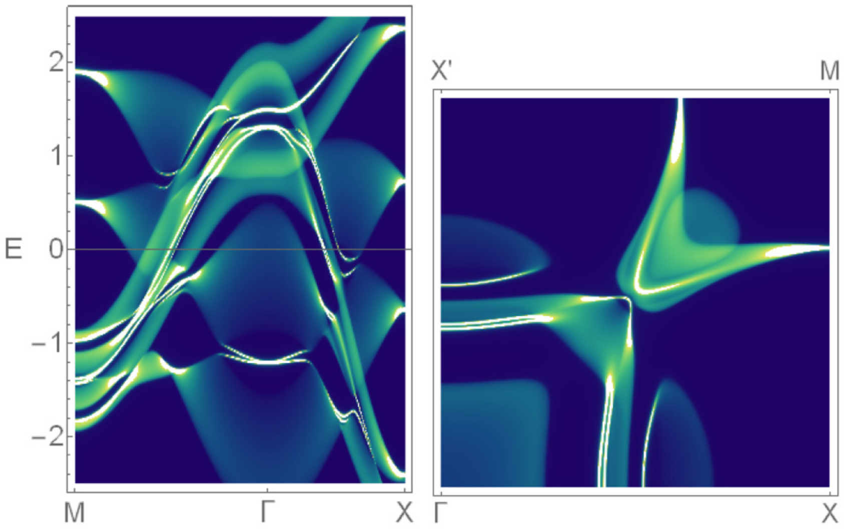

Supplementary Figure 8. *Left*: Surface spectral function $A_S(\boldsymbol{k}, E)$ showing the band structure for $\boldsymbol{k}$ along (M, Γ, X). *Right:* The same function at the Fermi level, $A_S(\boldsymbol{k}, 0)$ for $\boldsymbol{k}$ in one quarter of the first Brillouin zone.

2.4. Continuum model for the p_r bands

To construct a minimal continuum model that describes the p_r bands with strong Rashba spin-orbit locking we rotate to a basis which is cylindrical in the orbital wavefunctions (p_z, p_r, p_θ). We begin by expanding our Hamiltonian to second order around Γ and then noticing that the Hamiltonian becomes block-diagonal by choosing $\psi_s = (|p_z \uparrow -\rangle, |p_z \downarrow +\rangle, |p_r \uparrow +\rangle, |p_r \downarrow -\rangle, |p_\theta \uparrow +\rangle, |p_\theta \downarrow -\rangle)$:

$$H\Psi = \begin{pmatrix} H_s & 0 \\ 0 & H_s \end{pmatrix}\begin{pmatrix} \psi_s \\ s_x\psi_s \end{pmatrix}, \quad \text{(S13)}$$

where $|p_i s \pm\rangle = |p_i s, \sigma = 1\rangle \pm |p_i s, \sigma = 2\rangle$. To further simply the problem, we eliminate the p_θ and p_z bands using the Löwdin-Feshbach method[7]. The resulting 4×4 matrix is block-diagonal. The top 2×2 block in the basis $(|p_r \uparrow +\rangle, |p_r \downarrow -\rangle)$ is given by

$$H_r = \begin{pmatrix} \frac{k^2}{2m_+} + \beta_+ k^2 \cos(4\theta) - \mu + \epsilon & ie^{-i\theta} k\alpha \\ -ie^{i\theta} k\alpha & \frac{k^2}{2m_-} + \beta_- k^2 \cos(4\theta) - \mu - \epsilon \end{pmatrix}. \quad \text{(S14)}$$

The parameters $m_\pm$, $\beta_\pm$, μ, ϵ and α are independent of k and are related in complex ways to the original parameters, while θ is the angle between the vector $\boldsymbol{k}$ and the k_x direction.

We further simplify the Hamiltonian by assuming that the Fermi surface warping terms $\beta_\pm$ are not significant and can be ignored. Furthermore, we also replace the difference between $\frac{k^2}{2m+}$ and $\frac{k^2}{2m-}$ by a constant that we absorb into ϵ, such that the separation of the two Fermi surfaces is preserved. We then recover the Rashba bilayer Hamiltonian[8], which in the familiar basis $(|\uparrow, \sigma = 1\rangle, |\downarrow, \sigma = 1\rangle, |\uparrow, \sigma = 2\rangle, |\downarrow, \sigma = 2\rangle)$ can be written as

$$H_r(k) = \frac{k^2}{2m} - \mu - \epsilon\sigma_x + \alpha(k_x s_y - k_y s_x)\sigma_z. \quad \text{(S15)}$$

The eigenvectors obtained from this model have spin locked to momentum. The resulting spin textures in the normal state reproduce those existing in the literature[1,2,9]. The model (S15) is also simple enough to solve the Gorkov gap equations.

2.5. Zeeman splitting and pairing potentials

In the presence of a weak magnetic field taken along the x-direction, the Hamiltonian takes the form

$$H_r(k) = \frac{k^2}{2m} - \mu - \epsilon\sigma_x + \alpha(k_x s_y - k_y s_x)\sigma_z - h s_x, \quad \text{(S16)}$$

as stated in equation (7) in the main text. We note that we include the magnetic field only as a Zeeman effect, and we ignore orbital effects. The in-plane field breaks the spin degeneracy on each of the two p_r bands, giving rise to four bands with dispersions

$$E(\boldsymbol{k}) = tk^2 - \mu \pm \sqrt{\epsilon^2 + \alpha^2 k^2 + h^2 \pm 2h\sqrt{\epsilon^2 + \alpha^2 k_y^2}}, \quad \text{(S17)}$$

where $t = \frac{1}{2m}$. Clearly, the energy splitting is largest in the k_y direction, which is orthogonal to the magnetic field.

To introduce superconductivity, we consider a Hubbard density-density interaction with two terms, intra-sublayer U, and inter-sublayer, V. The U term can only induce spin singlet pairing, while the V term can induce both singlet and triplet pairings. As we expect U to dominate singlet pairing, we will only consider the spin-triplet component of V. This leaves the simplified interaction,

$$H_{\text{int}} = \int d\boldsymbol{k} d\boldsymbol{k}' \left[U \sum_{i,s} c^\dagger_{is\boldsymbol{k}} c^\dagger_{i\bar{s}-\boldsymbol{k}} c_{i\bar{s}-\boldsymbol{k}'} c_{is\boldsymbol{k}'} - V \sum_{i,s} c^\dagger_{is\boldsymbol{k}} c^\dagger_{\bar{i}s-\boldsymbol{k}} c_{\bar{i}s-\boldsymbol{k}'} c_{is\boldsymbol{k}'} \right], \quad \text{(S18)}$$

where i and s are sublattice and spin indices, respectively, and bars indicate the opposite value of the index. These interactions can induce four possible pairing potentials $\Delta_1 \propto I$, $\Delta_2 \propto \sigma_z$, $\Delta_3 \propto \sigma_y s_x$ and $\Delta_4 \propto \sigma_y s_y$.

To proceed analytically, we make use of the fact that Cooper pairing occurs primarily at the Fermi surface, and that Fermi surfaces of different bands do not mix. This allows us to treat each Fermi surface separately, projecting onto the electron and hole parts of the Hamiltonian. To be conservative, the Zeeman energy associated with the critical field of β-$PdBi_2$ is taken to be of the order of the superconducting gap, i.e., ~ 1meV. Therefore, the magnetic fields relevant to our calculation are much smaller than all parameters of the normal Hamiltonian, which are of the order of 1eV, so we treat the Fermi surfaces as only weakly split by the field. We construct and solve a 2 × 2 gap equation for each Fermi surface.

The problem thus becomes analytically tractable with the caveat that in the electron-hole basis the pairing potentials have a more complicated $\boldsymbol{k}$-dependent structure. The Bogoliubov-de-Gennes Hamiltonian takes the form

$$H_{\mathrm{BdG}}^{(\pm)} = \xi_k^{(\pm)} \tau_z - h \sqrt{\frac{1 + \rho_k^2 \sin^2(\theta)}{1 + \rho_k^2}} s_z \tau_0 + \Delta_{\boldsymbol{k}} \tau_x, \tag{S19}$$

where $\xi_k^{(\pm)} = t k^2 - \mu \pm \epsilon \sqrt{1 + \rho_k^2}$, $\rho_k = \alpha k/\epsilon$, $\Delta_{\boldsymbol{k}}$ contains both singlet and triplet pairings. The Pauli matrices τ_x, τ_y and τ_z act on the particle-hole degree of freedom. After projecting each pair potential in the band basis, we found that only pairing states of the same spin around the y-direction, $\sigma_y s_y$, have significant weight. We therefore consider the following two-component order parameter:

$$\Delta = \psi + \eta \sigma_y s_y. \tag{S20}$$

Supplementary references

1. Sakano, M. *et al.* Topologically protected surface states in a centrosymmetric superconductor β-$PdBi_2$. *Nature Commun.* **6**, 8595 (2015).
2. Iwaya, K. *et al.* Full-gap superconductivity in spin-polarised surface states of topological semimetal β-$PdBi_2$. *Nature Commun.* **8**, 976 (2017).
3. Shein, I. R. & Ivanovskii, A. L. Electronic band structure and Fermi surface of tetragonal low-temperature superconductor Bi_2Pd as predicted from first principles. *J Supercond Nov Magn* **26**, 1–4 (2013).
4. Tu, X.-H. *et al.* Topological superconductivity in Rashba spin-orbital coupling suppressed monolayer β-Bi_2Pd. *Materials Today Physics* **24**, 100674 (2022).
5. Sancho, M. P. L., Sancho, J. M. L., Sancho, J. M. L. & Rubio, J. Highly convergent schemes for the calculation of bulk and surface Green functions. *J. Phys. F: Met. Phys.* **15**, 851 (1985).
6. Wang, B. T. & Margine, E. R. Evolution of the topologically protected surface states in superconductor β-Bi_2Pd from the three-dimensional to the two-dimensional limit. *J. Phys.: Condens. Matter* **29**, 325501 (2017).
7. Löwdin, P.-O. A Note on the quantum-mechanical perturbation theory. *J. Chem. Phys.* **19**, 1396–1401 (1951).
8. Nakosai, S., Tanaka, Y. & Nagaosa, N. Topological superconductivity in bilayer Rashba system. *Phys. Rev. Lett.* **108**, 147003 (2012).
9. Xu, T. *et al.* Nonhelical spin texture in the normal states of the centrosymmetric superconductor β-$PdBi_2$. *Phys. Rev. B* **100**, 161109 (2019).
10. Gor'Kov, L. On the energy spectrum of superconductors. *Sov. Phys. JETP* **7**, 158 (1958).
